# Identification of primary thyroid lymphoma with medical imaging: A case report and review of the literature

**DOI:** 10.3892/ol.2014.2542

**Published:** 2014-09-17

**Authors:** JIA-HUAN WANG, LIANG CHEN, KE REN

**Affiliations:** 1 Department of Radiology, The First Hospital of Jilin University, Changchun, Jilin 130021, P.R. China; 2 Department of Radiology, Jilin Central Hospital, Jilin City, Jilin 132000, P.R. China; 3 Department of Radiology, The First Hospital of China Medical University, Shenyang, Liaoning 110001, P.R. China

**Keywords:** primary thyroid lymphoma, computed tomography, imaging features

## Abstract

Primary thyroid lymphoma (PTL) is a rare thyroid malignancy. Clinical diagnosis of PTL may not be easily established based on imaging studies, as the imaging features of PTL are similar to those of lymphocytic thyroiditis and primary thyroid cancer. The present study describes the case of a patient who was confirmed to have PTL by intra-operative pathological diagnosis. On color Doppler ultrasound, the PTL was shown as a significantly enlarged thyroid with reduced gland echoes. Color Doppler flow imaging showed increased blood flow. By computed tomography, the thyroid was revealed to be enlarged with reduced tissue density, particularly in the left lobe and the isthmus. In addition, calcified spots and swollen lymph nodes were evident. The clinical history of the patient was obtained and the imaging results were retrospectively analyzed. The imaging features of PTL were investigated through reviewing the literature. PTL exhibits specific features on medical imaging that aid in distinguishing it from other thyroid diseases. PTL exhibits specific features on medical imaging that aid in distinguishing PTL from other thyroid diseases, which may aid the support for clinical diagnosis and improve the clinical accuracy.

## Introduction

Lymphoma is malignancy of the lymph nodes or lymphatic tissue and is classified as either Hodgkin’s lymphoma or non-Hodgkin’s lymphoma (NHL). Primary lymphoma lesions are found in lymph nodes, as well as in the other tissues and organs, such as the thyroid, tonsil, nasopharynx, gastrointestinal tract and spleen. Typically, the affected lymph nodes are painless and progressively swollen. Primary thyroid lymphoma (PTL) is a rare extranodal lymphoma accounting for 2% of extranodal lymphomas (1), the majority of which are NHL. PTL often occurs among women 50–70 years old (2), and it is believed that ≥75% of patients with PTL have a history of lymphocytic thyroiditis. PTL is also associated with Hashimoto’s disease (3,4).

PTL exhibits complicated and diverse imaging characteristics, making it difficult to differentially diagnose from other thyroid diseases (5,6). Therefore, the clinical diagnosis primarily depends upon medical history and laboratory tests. However, the imaging studies of primary thyroid lymphoma were rarely applied in the past. In the current study, we present the case of a patient with PTL and review the relevant literature in order to summarize the imaging features of PTL. Patient provided written informed consent.

## Case report

### Case presentation

A female patient (58 years old) presented to the Jilin Central Hospital (Jilin, China) with a front neck mass and developing dysphagia. The mass progressed slowly for one and half years without treatment. The patient had no history of tobacco smoking or family history of tumor or thyroid disease. The front neck mass was 7.0×8.0 cm in size, firm and palpable with a larger portion on the left side, and moved during swallowing. The thyroid auscultation showed no vascular bruit. The thyroid function test results were consistent with lymphocytic thyroiditis: Thyroxine levels, 11.33 pmol/l (normal range, 12–22 pmol/l); triiodothyronine levels, 4.05 pmol/l (normal range, 3.1–6.8 pmol/l); and thyroid stimulating hormone levels, 4.98 μIU/ml (normal range, 0.27–4.2 μIU/ml). Thus, the initial diagnosis was lymphocytic thyroiditis and the patient was managed accordingly. The patient and her relatives were informed of the possibility of malignancy and that surgery should be performed if the mass was malignant or it compressed the neck.

### Imaging studies

**Figure 1:**
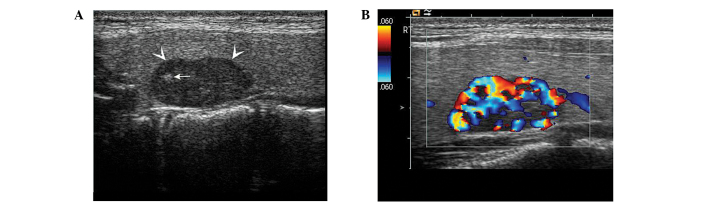
Color Doppler ultrasound demonstrating (A) non-uniform hypoechoic signals and an indication of calcified spots (white arrows). (B) Color Doppler flow imaging indicted increased blood flow signals.

**Figure 2:**
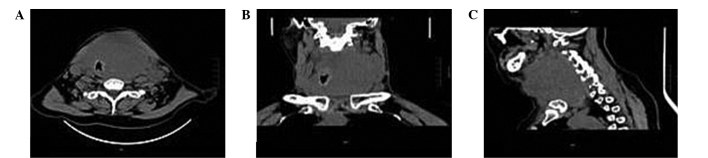
Multi-planar reconstruction computed tomography image of the neck shows a significantly enlarged thyroid, particularly in the left lobe and the isthmus, with reduced tissue density. The lesion extends from the upper level of the third cervical vertebra to the arterial arch, and presses the surrounding structures toward the right side. (A) Transversal view, (B) coronal view and (C) sagittal view.

A neck color Doppler ultrasound showed a significantly enlarged left lobe and isthmus of the thyroid (Fig. 1). The gland was non-uniformly hypoechoic, with an indication of calcified spots. Also, the trachea was pressed to the right. Color Doppler flow imaging showed increased blood flow signals. Computed tomography (CT) in the transverse view (Fig. 2A) revealed a significantly enlarged thyroid, particularly in the left lobe and isthmus, with reduced thyroid tissue density and calcified spots. The coronal and sagittal CT views (Fig. 2B and C) showed that the lesion extended from the upper level of the third cervical vertebra to the arterial arch. The largest lesion section was approximately 8.5×8.7 cm, with a longitudinal dimension of ~9.4 cm. The trachea, esophagus and soft tissues in the left neck were under pressure and displaced toward the right side. Soft tissue density shadows of 0.7–3.0 cm in diameter were observed medial to the sternocleidomastoid muscle. Enlarged abdominal and retroperitoneal lymph nodes were not detected on the abdominal CT image.

### Surgery and postoperative pathological examination

**Figure 3:**
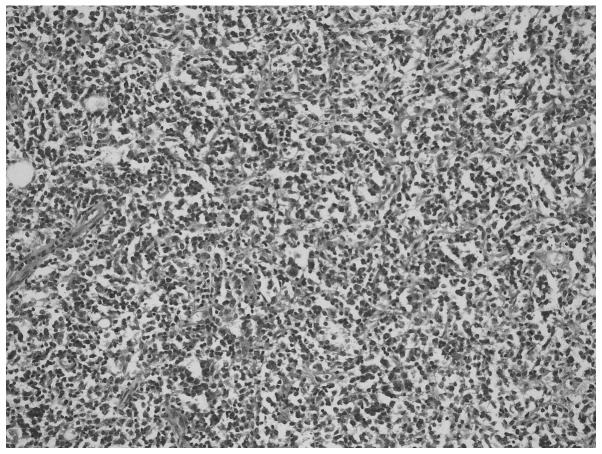
Pathological confirmation of non-Hodgkin’s lymphoma by diffuse proliferation of large atypical lymphocytes, large nuclei with coarse nuclear reticulum, including several small nucleoli, and the appearance of mitosis (staining, hematoxylin and eosin; magnification, ×20).

Surgery was performed one month after the patient’s initial visit, as the patient experienced worsened dysphagia. During surgery, a 9.0×12.0-cm firm tumor was observed to adhere to the cervical muscle, with a fish-flesh appearance and no complete capsule. Rapid intraoperative pathological diagnosis showed thyroid malignancy, thus lymphoma was indicated. Partial resection was performed due to difficulties in complete surgical resection. Immunohistochemical analysis showed that antigens associated with B cells, such as cluster of differentiation 20 (CD20), CD22 and CD79a (7–9), were widely expressed, suggesting B-cell lymphoma. Postoperative pathological examination (Fig. 3) confirmed diffuse large B-cell lymphoma by the diffuse proliferation of large atypical lymphocytes, large nuclei with coarse nuclear reticulum, including several small nucleoli and the appearance of mitosis.

### Chemotherapy and follow-up

Following surgery, five cycles of cyclophosphamide (750 mg/m^2^ by intravenous injection on day one), doxorubicin (50 mg/m^2^ by intravenous injection on day one), vincristine (1.4 mg/m^2^ by intravenous injection on day one), prednisone (100 mg/m^2^ by oral administration on the days one to five) and etoposide (40 mg/m^2^ by intravenous injection on days one to four) (CHOEP) chemotherapy was administered every three weeks for one cycle, during which bone marrow aspiration showed active proliferation of nucleated cells with no morphological abnormalities, suggesting bone marrow invasion. The postoperative CT image showed a 1.7×2.0-cm oval low-density mass anterior to the spleen, with 21–36 CT numbers. An enlarged lymph node 1.4 cm in diameter was observed posterior to the spleen and anterior to the inferior vena cava. The lymphoma concurred in the thyroid gland and spleen, with the involvement of lymph nodes on both sides of the diaphragm, thus it was staged as IIISE according to Ann Arbor stage (1971) (10,11).

Following nine cycles of CHOEP chemotherapy, the neck mass was not palpable and the splenic lesion decreased by 50%. The neck was bilaterally symmetric on follow-up ultrasonography. The lymph nodes decreased significantly and were soft upon palpation with good mobility. Follow-up was performed every six months for three years and at present, the patient has a good physical condition.

## Discussion

PTL lesions may involve the hypopharynx, esophagus or ipsilateral sternocleidomastoid muscle, causing symptoms such as hoarseness and breathing difficulties, and resulting in normal or reduced thyroid function. In total, 65–80% of PTL patients are estimated to test positive for thyroglobulin antibody and/or thyroglobulin monoclonal antibody in peripheral blood. In laboratory tests, PTL exhibits widespread expression of B-cell antigens. Autoimmune lymphocytic thyroiditis is present in the majority of PTL patients (12), but cytokines, particularly interleukin 7 (IL-7), play a key role in lymphoma. The high expression of IL-7 may be used in differentiating thyroid lymphoma from lymphocytic thyroiditis (13).

Multi-directional multi-point fine needle aspiration cytology is the primary diagnostic procedure for PTL, and it should be performed under ultrasound guidance. Alternatively, non-invasive medical imaging procedures are increasingly used in the diagnosis of thyroid malignancies. PTL images show complex features and can be easily mistaken for primary thyroid cancer or lymphocytic thyroiditis, as all three can exhibit asymmetric diffuse thyroid enlargement. The features of PTL in various imaging modalities are crucial in establishing the diagnosis of PTL by perceiving different aspects of the lesions, which may complement each other (14).

In ultrasound imaging, PTL exhibits a hypoechoic mass with a mixed echo structure. Calcification is uncommon and there is no liquefaction. Although the uninvolved thyroid tissues also exhibit low echoes, they have clear boundaries with extremely low echo areas of PTL, due to the presence of lymphocytic thyroiditis (12). In the present case, uneven distribution of reduced echoes in color Doppler ultrasound was similar to that reported in the literature (12), but calcification was observed.

On magnetic resonance imaging (MRI), PTL exhibits iso- or hyperintensities in T1- and T2-weighted images, with a linear low-signal separation observed in certain lesions, which indicate fiber separation (pseudocapsules) (15). Since MRI has a high contrast resolution for soft tissues, it can clearly show the uninvolved thyroid tissue. Thus, MRI is advantageous in the detection of pseudocapsules and the uninvolved thyroid tissues. In nuclear medicine, single photon emission computed tomography shows cold thyroid nodules or a reduced intake area in PTL patients. Positron emission tomography (PET) or PET/CT imaging is now employed in patients with rising thyroglobulin levels, while other imaging modalities, such as ultrasound imaging, CT and MRI, fail to reveal the sites of disease (16,17).

CT imaging of PTL often shows homogeneous and symmetric enlargement of the lobes of the thyroid gland and isthmus with densities lower than the adjacent muscles (18). With an enhanced image, the gland density is evenly enhanced without showing lower-density nodules or calcification (19). When PTL occurs in a unilateral lobe or in a unilateral lobe plus thyroid isthmus, in CT images it often protrudes from the thyroid capsule with unclear borders. By contrast, a normal thyroid appears as a narrow strip under pressure. The contralateral lobe density is often uneven and, following enhancement, the heterogeneity is increased; however, no obvious mass is observed, which may be due to autoimmune thyroiditis in PTL patients (18). When PTL occurs in bilateral lobes as well as the isthmus, lymphocytic thyroiditis often occurs simultaneously, and the borders are clearer (21). In contrast-enhanced CT (CECT) images, lesions are mildly to moderately enhanced with CT numbers increasing by 15–20 HU, but the degree of enhancement remains lower than that of the adjacent muscle.

There are imaging features that identify PTL from other thyroid diseases. On CT scans, thyroid cancer exhibits uneven mass density and diffuse gland enlargement with partial cystic formation, which can contain mural nodules. These nodules are enhanced in CECT scans, and the ‘peninsula-like’ nodules are a specific feature in the diagnosis of thyroid cancer (18,21). Thyroid adenoma has clearer borders, and thus is easier to identify (21). Diffuse goiter has a diverse and complex performance on images. The thyroid leaves and isthmus swell symmetrically, with reduced or very uneven densities, showing a wide range of cystic change and calcification, but no lymph node enlargement (22).

In conclusion, three aspects should be considered for the accurate diagnosis of PTL; clinical examination, laboratory test results and imaging studies. For elderly female patients presenting with front neck masses, consideration of the possibility of PTL should combine the history of lymphocytic thyroiditis, laboratory test results and specific imaging features.
